# Transcranial Magnetic Stimulation Combined With Electroencephalography Analysis Suggest Time‐Dependent and Region‐Specific Modulation of Cortical Excitability During Acute Alcohol Intoxication

**DOI:** 10.1111/acer.70407

**Published:** 2026-09-05

**Authors:** Virve Kekkonen, Evita Fonsén, Elisa Kallioniemi, Laura Säisänen, Tommi Tolmunen, Mervi Könönen, Petri Kivimäki, Sara Määttä

**Affiliations:** ^1^ Department of Adolescent Psychiatry Kuopio University Hospital Kuopio Finland; ^2^ Adolescent Psychiatry, Institute of Clinical Medicine University of Eastern Finland Kuopio Finland; ^3^ University of Helsinki and HUS Helsinki University Hospital Helsinki Finland; ^4^ Terveystalo Seinäjoki Finland; ^5^ Department of Biomedical Engineering New Jersey Institute of Technology Newark New Jersey USA; ^6^ Neurology, Institute of Clinical Medicine University of Eastern Finland Kuopio Finland; ^7^ Neuro Center Kuopio University Hospital Kuopio Finland; ^8^ Department of Clinical Radiology Kuopio University Hospital Kuopio Finland; ^9^ Radiology, Institute of Clinical Medicine University of Eastern Finland Kuopio Finland; ^10^ Malmi Psychiatric Outpatient Clinic, City of Helsinki Helsinki Finland; ^11^ Clinical Neurophysiology, Institute of Clinical Medicine University of Eastern Finland Kuopio Finland; ^12^ Department of Clinical Neurophysiology Kuopio University Hospital Kuopio Finland

**Keywords:** adolescence, adulthood, alcohol, cortex, intoxication, TMS‐EEG

## Abstract

**Background:**

Longitudinal excessive alcohol consumption has adverse effects on brain structure and functions. The immediate effects of alcohol consumption on the activity and functioning of different circuits in the brain are less known. This exploratory study aimed to examine the effects of acute alcohol exposure on cortical activity in healthy adults.

**Methods:**

The study included 34 adults, aged 29–36 years, who took part in the Youth and Alcohol Project's (YAP) alcohol exposure test. The participants' alcohol consumption was measured with the shortened version of the Alcohol Use Disorders Identification Test (AUDIT‐C). Cortical functioning was investigated at baseline (BL) before alcohol intoxication, and 30 min (T2) and 120 min (T3) after alcohol intoxication using navigated transcranial magnetic stimulation combined with electroencephalography (TMS‐EEG), focusing on TMS‐evoked EEG potential (TEP) peaks P30, N45, P60, N100, and P180. The blood alcohol level was monitored using an alcohol breathalyzer.

**Results:**

During alcohol intoxication, there were changes in the global mean field power (GMFP) and TEP magnitudes compared to BL. After alcohol consumption, the global mean field power (GMFP) amplitude decreased from approximately 80 ms poststimulus onward. For P30, N45, P60, and P180, the mean amplitude decreased in T2 compared to BL and recovered in T3. For N100, the mean amplitude decreased in T2 and in T3. Differences in TEP peaks between BL and T2 or BL and T3 were emphasized in frontal and central brain regions.

**Conclusions:**

The results suggest time‐dependent and region‐specific modulation of cortical excitability during acute alcohol intoxication. Alcohol's effects are dynamic, regionally heterogeneous, and not limited to simple global suppression. TMS‐EEG offers a promising tool for assessing alcohol‐related changes in cortical functions.

## Graphical abstract

This study examined how acute alcohol exposure affects brain cortical activity in 34 healthy adults using TMS‐EEG. Measurements were taken before, 30 min after, and 120 min after intoxication. Alcohol altered brain activity in a time‐dependent manner, reducing signal amplitudes shortly after intake, with partial recovery later. Effects varied across brain regions, especially frontal and central areas. Findings indicate that alcohol's impact on cortical excitability is dynamic and region‐specific rather than uniformly suppressive.

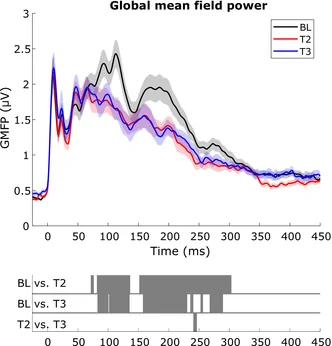

## 1 Introduction

### 1.1 Alcohol's Effects on the Brain

Heavy alcohol use is known for its detrimental effects on cognitive functioning and association with alterations in cerebral gray and white matter volumes (Heikkinen et al. 2017; Karoly et al. 2024; Squeglia and Gray 2016). There is also increasingly more research on how acute alcohol intoxication has detrimental effects on parameters associated with executive functions, such as response inhibition (McPhee and Hendershot 2023), and performance in working memory tasks (Spinola et al. 2022). Acute alcohol intoxication is characterized by reductions in electroencephalogram (EEG)‐derived event‐related potentials (ERPs), components linked with attention, automatic auditory processing, and performance monitoring (Fairbairn et al. 2021).

### 1.2 TMS‐EEG in Alcohol Research

Transcranial magnetic stimulation (TMS) is a noninvasive and painless method for stimulating cortical neurons (Barker et al. 1985). When combined with simultaneous electroencephalography (EEG), it evolves into a sophisticated method known as TMS‐EEG, capable of exploring the neurophysiological characteristics and brain dynamics of any neocortical brain area or network (Ilmoniemi and Kičić 2010). TMS‐EEG offers an in vivo evaluation of the excitability of the human cortex and effective connectivity (Chung et al. 2015; Ilmoniemi and Kičić 2010; Paus et al. 2001). The technique provides results that are distinct from other functional brain imaging techniques and is particularly well suited for alcohol research. This is because alcohol affects the brain through neurotransmitters, including gamma amino butyric acid (GABA) and glutamate receptor activity (Hillmer et al. 2015; Montemitro et al. 2025), and the TMS‐evoked EEG potentials (TEPs) N45, P60, and N100 are known to be linked to GABA‐A‐, GABA‐B‐, and glutamate receptor‐mediated neurotransmission (Belardinelli et al. 2021; Du et al. 2018; Ferreri et al. 2011; Gordon et al. 2023; Hillmer et al. 2015; Premoli, Castellanos, et al. 2014). Unlike passive EEG or ERP paradigms, which measure spontaneous or stimulus‐driven cortical activity, TMS‐EEG enables causal perturbation of defined cortical circuits and quantification of the resulting evoked responses, providing a direct readout of cortical excitability and effective connectivity (Hernandez‐Pavon et al. 2023; Ziemann et al. 2026). This perturbation‐based approach is also complementary to neuroimaging modalities such as functional magnetic resonance imaging (fMRI), which captures hemodynamic correlates of neural activity without neurotransmitter specificity and positron emission tomography (PET) imaging, which captures receptor activity but lacks temporal resolution. TMS‐EEG offers millisecond‐level temporal precision combined with sensitivity to specific receptor‐mediated processes (Belardinelli et al. 2021; Premoli, Castellanos, et al. 2014; Premoli, Rivolta, et al. 2014). Because individual TEP components can be linked to distinct neurotransmitter systems, TMS‐EEG can function as a non‐invasive pharmacodynamic probe, potentially enabling identification of which inhibitory or excitatory mechanisms are most disrupted at specific intoxication stages. This capacity has direct translational relevance as TEP‐derived markers could inform the development of targeted pharmacological interventions for alcohol use disorder and serve as biomarkers for monitoring treatment response.

The fundamental principle of TMS‐EEG involves applying TMS to a target brain area, which instantaneously activates the neurons and generates TEPs. The waveform of TEPs partly depends on the stimulation site (Kähkönen et al. 2005). TMS of the motor cortex produces a sequence of TEP components or peaks at specific latencies and polarities, typically observed around 30 ms (P30), 40–50 ms (N45), 60–70 ms (P60), 80–120 ms (N100), 160–200 ms (P180), and 250–350 ms (N280) (Ferreri et al. 2011; Määttä et al. 2017; Rogasch et al. 2014). The amplitude and shape of these TEP peaks are significantly influenced by brain maturation and aging processes (Ferreri et al. 2017; Määttä et al. 2017; Noda et al. 2021; Opie et al. 2018). Sensory artifacts, such as the auditory responses elicited by the TMS click sound, are possible during stimulation and may impact the TEPs, specifically after 70–80 ms (Freedberg et al. 2020; Gordon et al. 2023).

### 1.3 Existing Evidence and Knowledge Gaps

The first study using TMS‐EEG on alcohol research reported that acute alcohol intake attenuated the global mean field amplitude following motor cortex stimulation, while later research (Kähkönen et al. 2003) revealed similar attenuation in response to prefrontal cortex stimulation. Furthermore, Kähkönen and Wilenius (2007) found that alcohol nearly abolished the N100 peak evoked by motor cortex stimulation. Consistently, alcohol has also been demonstrated to reduce the N100 amplitude following prefrontal cortex stimulation (Loheswaran et al. 2018). In addition, Loheswaran et al. (2017) reported that alcohol intoxication impaired paired associative stimulation (PAS)‐induced neuroplasticity in the left dorsolateral prefrontal cortex (DLPFC) and globally (Loheswaran et al. 2017). Furthermore, in the previous study phase of the Youth and Alcohol Project (YAP), a motor cortex TMS‐EEG study revealed a significant increase in the N45 peak and topographical alterations in the P60 and N100 peaks in those with a history of heavy drinking (Kaarre et al. 2018).

### 1.4 Study Rationale

To our knowledge, excitation and inhibition related neurochemistry corresponding to TMS‐EEG profiles in different alcohol intoxication stages remains incompletely defined, and previous TMS‐EEG studies on acute alcohol intoxication are limited. Nonetheless, existing research has demonstrated that acute alcohol exposure induces measurable neurophysiological changes in healthy individuals. Furthermore, none of these studies examined in detail the effects of alcohol on the early TEPs or how TEPs recover over time following alcohol consumption.

### 1.5 Aims of This Study

We aimed to investigate the impact of alcohol intoxication on neurophysiological functions in healthy adults using TMS‐EEG in an exploratory study. Additionally, we aimed to provide a more detailed understanding of how acute alcohol intoxication modulates cortical excitability over time.

## 2 Materials and Methods

### 2.1 Participants

**FIGURE 1:**
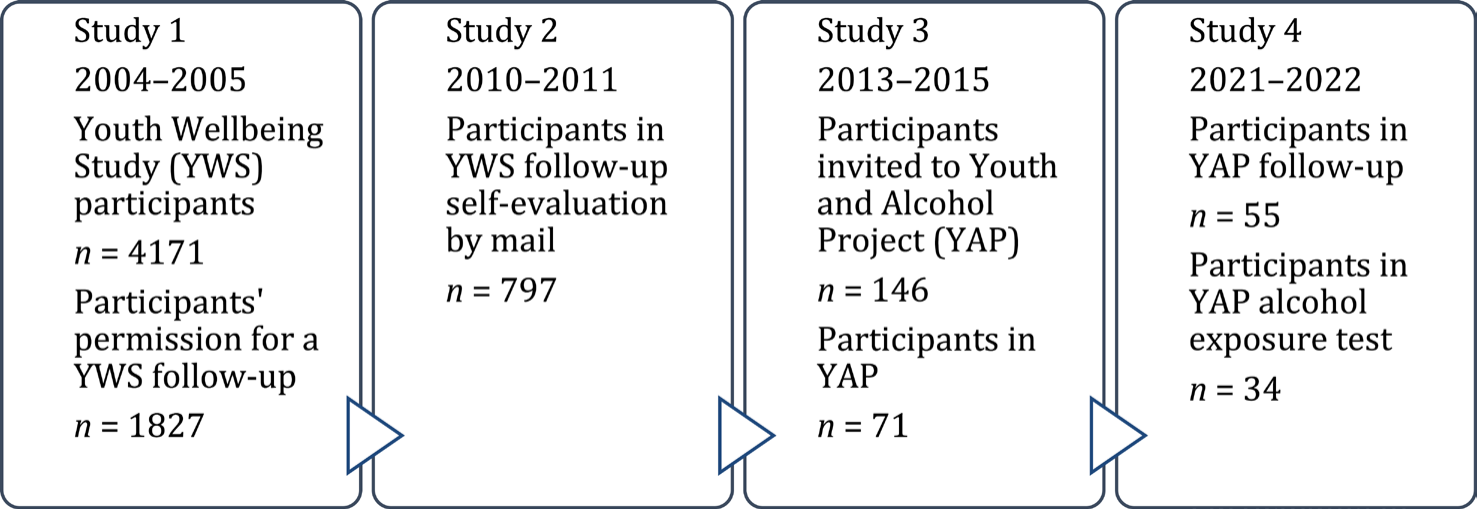
Timeline of the preceding studies and participants of the Youth and Alcohol Project during 2004–2022.

The Youth and Alcohol Project (YAP) study was conducted as an extension of the Youth Wellbeing Study (YWS) (Kekkonen et al. 2015; Laukkanen et al. 2009), which has longitudinally monitored the health and alcohol use of adolescents, as well as how long‐term alcohol use affects the developing brain. YAP study protocols have been presented earlier (Heikkinen et al. 2017; Kaarre et al. 2015). The YAP timeline and the participant recruitment process are presented in Figure 1.

Ethical approval for the study was granted by the Research Ethics Committee of Kuopio University Hospital (no. 323/20152027) and North Savo Wellbeing Services (no 31/2019). The experiments adhered to the latest iteration of the Helsinki Declaration, and all participants provided written informed consent. The exclusion criteria for neuroimaging methods were a history of a head injury needing medical treatment, neurological illness, a severe mental disorder, metal, or implanted devices in the body contraindicating magnetic resonance imaging (MRI), regular use of other intoxicating substances, and pregnancy.

The participants were chosen based on their alcohol use measured with the shortened consumption‐focused version of the Alcohol Use Disorders Identification Test (AUDIT) (Saunders et al. 1993), AUDIT‐C (Bush 1998). Eligibility for inclusion was determined by predefined criteria requiring consistent AUDIT‐C scores across both assessments (≥ 3 or ≤ 2 at both time points), while those with intermediate or inconsistent scores were not included in the analyses. The YAP protocol included MRI and fMRI of the brain, neurophysiological tests (TMS‐EEG), cognitive psychological tests, self‐rated questionnaires, and psychiatric interviews. There were no differences in neuropsychological test performance between the study groups (Kaarre et al. 2015).

The YAP neuroimaging follow‐up study conducted from January 2021 to November 2022 (Study 4, *n* = 55 (28 males, 27 females)) included participants aged 29–36 years. Eight participants had previously taken part only in the YWS, not the YAP. Of the initial cohort of 55 participants, one was excluded due to technical difficulties with electrode placement. The YAP neuroimaging follow‐up study included navigated TMS‐EEG, a psychiatric interview, and a self‐rated questionnaire on substance use. This resulted in a cohort of 54 participants, of whom 34 actively participated in the alcohol exposure test; only one individual refused to complete the T3 measurement of this test. Consequently, we successfully collected and analyzed data from 33 participants at each of the three measurement time points in the test, while the remaining participants underwent baseline (BL) measurements only.

### 2.2 Study Protocol Overview

In the morning, each participant attended a psychiatric interview, completed self‐rated questionnaires, and underwent structural brain imaging. The participants had lunch before the afternoon alcohol exposure test. This test, combined with the navigated TMS‐EEG design, was carefully executed at three time points: time point 1, the initial baseline (BL) measurement conducted before the onset of alcohol consumption, time point 2 (T2), 30 min after alcohol consumption, which was at the expected peak of the blood alcohol concentration (BAC), and time point 3 (T3), 120 min after alcohol consumption. This three‐timepoint approach allowed us to capture the dynamic shifts in brain responses associated with the start of acute alcohol intoxication and the potential adaptive effects that manifest later during the intoxication episode. At the end of the alcohol exposure test, just before T3, participants were offered snacks and a nonalcoholic beverage. Participant well‐being was monitored during the whole study protocol by trained clinicians.

### 2.3 Measurements

Before the alcohol exposure test, the quantity and frequency of alcohol consumption by the participants was assessed with the self‐rated AUDIT‐C questionnaire (Bush 1998). The participants' mental health and alcohol use disorders, such as alcohol abuse or dependence (APA 1994), were assessed with the Structured Clinical Interview for the 4th edition of the Diagnostic and Statistical Manual of Mental Disorders (DSM‐IV), Axis I (First et al. 1997) and II disorders (First et al. 1994), by a trained clinician. In addition, lifetime drug use and current smoking status were also recorded.

### 2.4 MRI

All subjects underwent a brain MRI session with a 3 T MRI scanner (Philips Achieva, The Netherlands). MRI sequences included 3D T1‐weighted, T2‐weighted, and Flair imaging. The T1‐weighted image was used for neuronavigation during TMS (acquired in the sagittal plane covering the whole brain, voxel size 0.94 mm × 0.94 mm × 1 mm, 190 contiguous slices, repetition time 8.24 ms, echo time 3.82 ms, flip angle 8°). Structural abnormalities were ruled out by an experienced neuroradiologist.

### 2.5 Alcohol Exposure Test

In the alcohol exposure test, the pharmacokinetics of alcohol (Jones et al. 1992) were considered when choosing a time window for neurophysiological tests. The target BAC was set at 1.0 permilles and monitored using a calibrated alcohol breathalyzer. Individually required amounts of alcohol for the target BAC were calculated by considering sex and body weight with the formula: Target BAC = 0.4 g/L = G × 100 × 0.8/(W × F), where G is the grams of alcohol, W is the body weight (in kilograms), and F is the conversion factor for the calculation of total body water (0.583 and 0.485 in male and female participants, respectively) (Fisher et al. 1987). After nine cases, we noticed that the evaluated BAC values remained too low, as the BAC mean was below 0.5 permilles. To correct the alcohol dose, the previous formula was multiplied by a factor of 1.15 from case 10 forward. We used Koskenkorva 38% alcohol (produced by Anora Group Plc) diluted with the participant's choice of sugared berry or fruit juice and preferred ratio for drinks. Drinks were consumed by the participants within around 15 min. The participants' BACs were measured repeatedly together with TMS‐EEG responses during the alcohol exposure test: at baseline before alcohol consumption, 30 min after alcohol consumption, and 120 min after alcohol consumption. BACs were tabulated at each time point. Additionally, each participant's peak BAC (the highest value across all time points) was recorded.

### 2.6 TMS‐EEG

Navigated TMS combined with high‐density TMS‐EEG measurements were conducted using a TMS‐compatible 64‐channel (61 EEG‐channels according to the 10–10 EEG system) EEG amplifier (NeurOneTesla EEG System, Bittium Biosignals Ltd., Finland) with a 20 kHz sampling frequency. The EEG system facilitated uninterrupted data recording without saturating the EEG signals and eliminated the need to maintain a constant output level during TMS by pinning the preamplifier. To mitigate electrode overheating caused by the stimulating coil, TMS‐compatible Ag‐/AgCl‐coated electrodes (EasyCap GmbH, Germany) were employed. Skin/electrode impedance was kept below 5 kΩ, and wire arrangements were adjusted prior to measurements to eliminate loops. The ground and reference electrodes were positioned on the forehead. Horizontal and vertical eye movements were monitored by recording the electro‐oculogram using two electrodes positioned adjacent to the external canthi, one on each side.

TMS was conducted using an eXimia stimulator (Nexstim Plc., Helsinki, Finland) coupled with a biphasic figure‐of‐eight coil, alongside the eXimia navigation system (version 4.3). This system allows continuous visualization of the stimulation site in relation to the individual's cortical anatomy. Muscle activity was monitored in real time and recorded using TMS‐locked electromyography (EMG) (Nexstim Plc., Helsinki, Finland). Motor evoked potentials (MEPs) induced by TMS were recorded with disposable Ag/AgCl surface electrodes placed on the right abductor pollicis brevis (APB) muscle using a belly‐tendon montage.

A reconstructed 3D brain surface, rendered at a depth where the gyral anatomy was distinguishable, was used to navigate the stimulation sites. To localize the motor representation of the right APB muscle, stimulation was performed around the anatomical “hand knob” (Yousry et al. 1997) with the induced current oriented perpendicular to the central sulcus. Mapping began at 50% of the maximal stimulator output, and intensity was then adjusted to elicit MEPs of 300–500 μV. The precentral gyrus and surrounding sulci were stimulated to identify the site where maximal MEP amplitudes were reliably evoked in the APB. At this optimal site, the coil was rotated within ±90° in the tangential plane to determine the most effective current direction. The location and orientation yielding the highest MEPs were designated as the stimulation target. Finally, the resting motor threshold (rMT, representing the minimum intensity of TMS needed to evoke an MEP) for the APB was determined at this target site using the TMS Motor Threshold Assessment Tool 2.0, with a minimum MEP amplitude criterion of ≥ 50 μV (Awiszus and Borckardt 2012).

Targets for all TMS recordings were defined at BL recording. The rMT was measured in each condition, but the baseline rMT was used for all stimulations. TMS was precisely directed toward the predetermined stimulation target at an intensity set to 90% of rMT. Each subject underwent 120 trials per condition, with interstimulus intervals randomized between 3 and 4 s. To mask the click sounds generated by the coil and prevent click‐induced auditory evoked potentials in the EEG, white noise derived from the TMS click waveform was digitized and processed into a continuous audio signal with specific time‐varying frequencies (Massimini et al. 2005). This white noise was continuously delivered through earplugs, with the masking volume adjusted until participants reported that the TMS click was no longer audible. A thin layer of foam was placed between the coil and the electrodes to reduce bone‐conducted sound and artifacts caused by electrode movement. Throughout the TMS‐EEG recording session, participants were seated in an adjustable chair with a headrest to ensure a stable head position. Participants were instructed to maintain their eyes open and fixate on a point displayed on a screen in front of them, aimed at minimizing ocular movements.

### 2.7 EEG Data Analysis

Offline data analysis was conducted with in‐house‐written scripts using MATLAB (version 2018a) and the freely available EEGLAB toolbox (Delorme and Makeig 2004) and was in accordance with previously published studies (Korhonen et al. 2011; Litvak et al. 2003; Rogasch et al. 2014; Saari et al. 2018) and TMS‐EEG toolboxes (Atluri et al. 2016; Rogasch et al. 2017). First, low‐frequency drift was filtered out from the raw EEG data with a 4‐s median filter. Next, the data were zero‐padded between 0 and 10 ms after each TMS pulse, and channels with poor contacts or excessive artifacts were removed. Then, the data were divided into segments (−1000 to 1000 ms around each TMS pulse) and baseline‐corrected using data between −1000 and 0 ms. Thereafter, independent component analysis (ICA) was run to remove the large‐amplitude TMS‐induced artifacts, and the resulting ICA weights were applied to continuous data to prevent edge effects in the filtering step. Independent components manually identified as artifactual were removed from the data. This was followed by interpolating the data between 0 and 15 ms after each TMS pulse, resampling the data to 1 kHz, and filtering the data with a 1–80 Hz bandpass and 50 Hz notch filter. The data were also visually checked, trial‐by‐trial, and channel‐by‐channel; largely artifactual trials were removed, and remaining poor channels were interpolated from neighboring channels. Finally, ICA was run on the data a second time to remove any remaining artifacts, such as blinks and muscle movements. Before proceeding with the analysis, the data were re‐referenced to the common average reference and baseline‐corrected a second time using data between −1000 and 0 ms.

The total EEG activity was first assessed using the global mean field power (GMFP) calculated as the root mean‐squared value of the EEG signal across all electrodes (Lehmann and Skrandies 1980). For the analysis of evoked responses, averaged TEPs over all the included trials for each electrode and each participant were used, and semi‐automatic amplitude/latency measurements of each TEP component were performed. Amplitude measurements followed a baseline‐to‐peak approach. In this study, to characterize primary motor cortex stimulation, we defined the TEPs in the same time frames as those used in our previous motor cortex study (Kaarre et al. 2018). P30 was assessed as the peak positivity occurring within the time frame of 20–40 ms, N45 as the maximum negative amplitude observed between 35 and 60 ms, P60 as the peak positivity occurring within the range of 50–90 ms, N100 as the peak negativity observed between 75 and 150 ms, and P180 as the maximum positivity observed between 160 and 200 ms.

**FIGURE 2:**
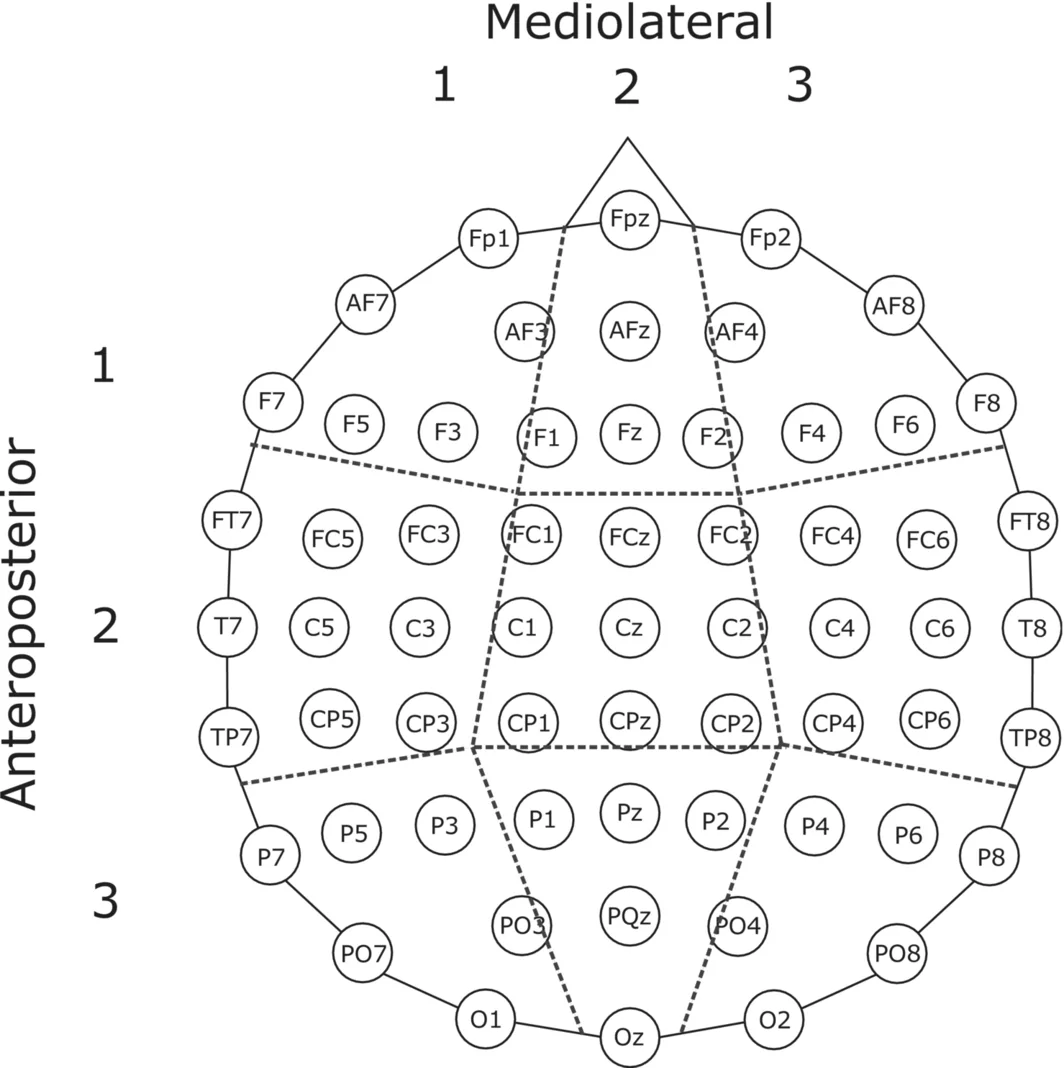
In the analysis of transcranial magnetic stimulation‐evoked potential peaks, we utilized a set of 61 electroencephalogram channels. These channels were categorized into nine regions based on their anteroposterior (AP) and mediolateral (ML) positioning.

When analyzing the topography of the TEP peaks, data from a total of 61 channels were utilized. These channels were categorized into nine regions based on their anteroposterior and mediolateral positioning: left frontal (LF), mid‐frontal (MF), right frontal (RF), left central (LC), mid‐central (MC), right central (RC), left posterior (LP), mid‐posterior (MP), and right posterior (RP), as depicted in Figure 2.

### 2.8 Statistical Analysis

Continuous variables were presented as means and standard deviations or standard errors, and categorical variables were presented as percentages and counts. AUDIT‐C scores were presented as the median and interquartile range (IQR).

During statistical analysis, differences in GMFP across BL, T2, and T3 were evaluated using paired *t*‐tests at each millisecond across the post‐TMS epoch. No correction for multiple testing was applied due to the exploratory nature of these analyses. Butterfly plots and topoplots were derived from electrode Cz at baseline timepoints (28, 44, 64, 112, and 188 ms) which were closest peaks to P30, N45, P60, N100, and P180.

We confirmed the normal distribution of rMTs at time points BL, T2, and T3. Subsequently, we conducted an ANOVA repeated measures test to assess the statistical significance of differences between rMTs across these time points.

TMS‐EEG data and 61 channels were reclassified to nine brain regions (Figure 2), which were reclassified as two three‐level categorical covariates according to their anteroposterior (1 = frontal, 2 = central, 3 = posterior) and mediolateral (1 = right, 2 = midline, 3 = left) locations. For each TEP component (P30, N45, P60, N100, and P180), a linear mixed model was fitted with time point (BL, T2, and T3) as a categorical fixed effect and participant as a random intercept. Anteroposterior position (frontal, central, posterior) and mediolateral position (left, midline, and right) were included as fixed effects. No interactions between time point and location were evaluated. Significant omnibus effects were followed by pairwise comparisons with no correction. No correction for multiple testing was applied due to the limited number of comparisons and the exploratory nature of these analyses.

Associations of background covariates (AUDIT‐C score, BAC levels (T2, T3, and peak of all BAC measurements during the alcohol exposure test), lifetime psychiatric diagnosis, smoking, substance or illegal drug use) to TEPs at each time point were investigated with linear mixed models for each brain region separately. Statistical calculations were conducted using SPSS version 29. *p*‐values below 0.05 were considered statistically significant.

## 3 Results

### 3.1 Characteristics of the Study Sample

The participants in the alcohol exposure test (*n* = 34) were 30–36 years old (mean 32.46; standard deviation 1.44) and 23 of them (65.7%) were males. The median score for AUDIT‐C was 3.00, range 0–8, and IQR was 4. The distribution of AUDIT‐C scores was positively skewed (skew = 0.350, SE (skew) = 0.398). All the time points of the alcohol exposure test were completed by all 33 participants.

According to the SCID‐I interviews, three participants had lifetime psychiatric diagnoses: the first participant was diagnosed with alcohol dependence (DSM‐IV 303.90), the second with dysthymic disorder (DSM‐IV 300.4), and the third with major depressive disorder (DSM‐IV 296.3), recurrent, mood disorder due to a general medical condition (DSM‐IV 293.83), obsessive‐compulsive disorder (DSM‐IV 300.3), and somatization disorder (DSM‐IV 300.81).

The mean (SD) BAC during the alcohol exposure test was 0.69 (0.20)‰ at T2 (approximately 30 min after alcohol consumption) and 0.58 (0.14)‰ at T3 (approximately 120 min after alcohol consumption), with a statistical difference between timepoints (*p* < 0.001). The mean (SD) for the peak of all BAC measurements during the alcohol exposure test was 0.72 (0.20)‰.

The mean (SD) rMT was 47.29 (10.32) at BL, 47.65 (9.56) at T2, and 48.16 (10.58) at T3. Paired sample testing revealed no significant differences in rMT among time points. Consequently, the observed changes in EEG responses cannot be attributed to variations in rMT.

### 3.2 Global Mean Field Power

**FIGURE 3:**
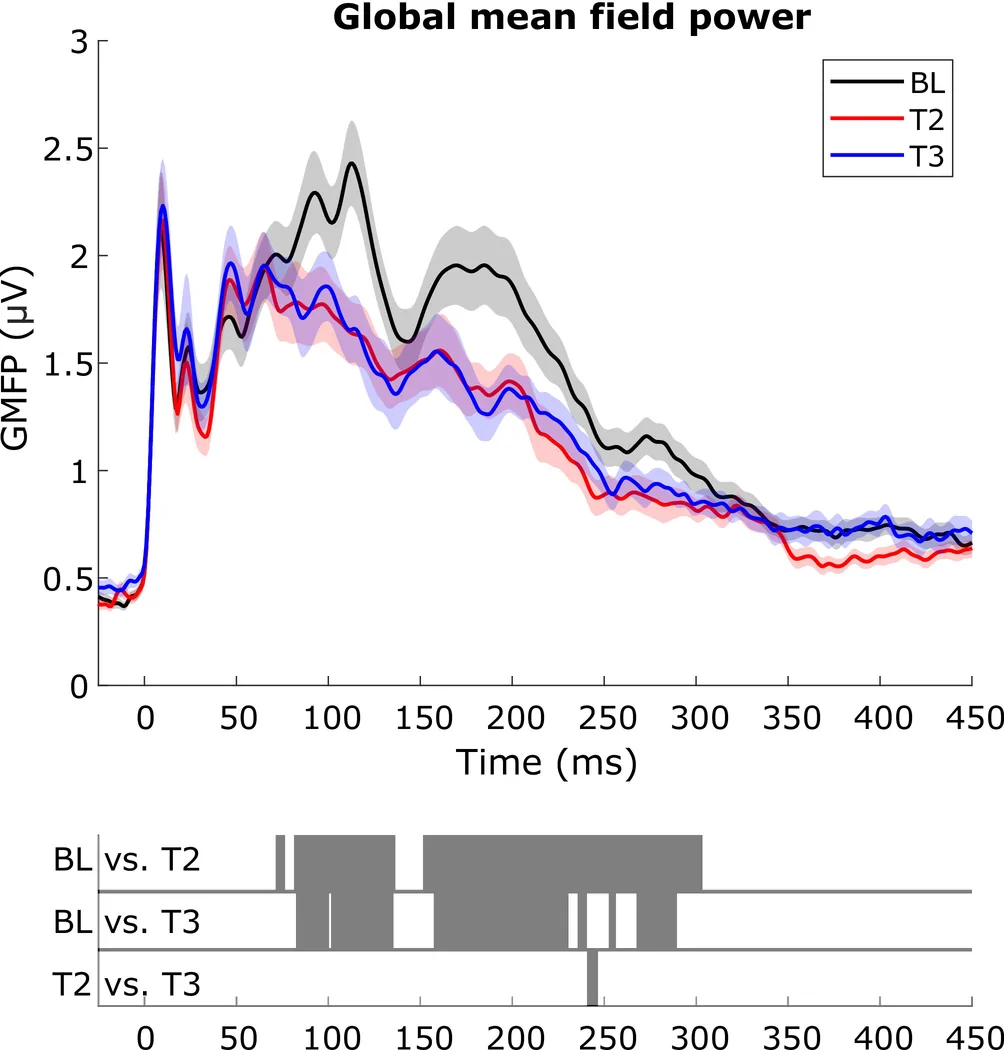
The grand average of global mean field power (GMFP) (μV) presented over time (ms) at BL (black line), T2 (red line), and T3 (blue line). Standard deviations are marked with shaded areas. Significant differences between groups are presented in the lower panel. Data are presented from 33 participants who completed all time points (BL, T2, T3).

Overall activation of the brain measured with GMFP decreased between BL and T2, as well as between BL and T3, in the respective time windows of 72–303 and 83–289 ms post‐TMS (Figure 3).

### 3.3 TEP Amplitudes

Global TEP amplitudes across all brain regions are presented in Table 1. Among the five TEP components, only N100 showed a significant (*p* < 0.001) omnibus effect of time point (BL, T2, and T3). Post hoc comparisons revealed that N100 amplitude was significantly attenuated (less negative) at both T2 (*p* < 0.001) and T3 (*p* < 0.001) relative to BL, with no significant change between T2 and T3 (*p* = 0.669), indicating a sustained reduction that did not recover within the measurement period. For P60, although the omnibus test did not reach significance (*p* = 0.085), the pairwise comparison between BL and T2 was significant (*p* = 0.027). No statistically significant effects were observed for P30, N45, or P180 at the global level.

**TABLE 1:** Mean amplitudes, standard errors (SE), and *p*‐values for the entire brain (overall) at three time points (BL, T2, and T3) and at five TEP peaks (P30, N45, P60, N100, and P180). The sign prior to the value indicates the polarity of TEP, + means positive and − means negative.

Amplitude	BL	T2	T3	*F*	Overall
	Mean (SE)	Mean (SE)	Mean (SE)		*p*
P30 (μV)	1.42 (0.15)	1.32 (0.15)	1.37 (0.15)	0.663	0.515
N45 (μV)	−1.55 (0.14)	−1.39 (0.14)	−1.45 (0.14)	1.197	0.303
P60 (μV)	2.08 (0.18)	1.78 (0.18)	1.79 (0.18)	2.473	0.085
N100 (μV)	−2.61 (0.22)	−2.04 (0.22)	−1.93 (0.22)	11.880	< 0.001
P180 (μV)	0.93 (0.09)	0.79 (0.09)	0.82 (0.09)	0.772	0.486
**Amplitude**	**BL vs. T2**				
	**Mean difference (μV) (SE)**	**95% CI for difference**	***p***		
P30 (μV)	0.101 (0.111)	−0.117 to 0.318	0.363		
N45 (μV)	−0.156 (0.110)	−0.373 to 0.060	0.157		
P60 (μV)	0.294 (0.133)	0.033–0.555	0.027		
N100 (μV)	−0.576 (0.130)	−0.831 to −0.321	< 0.001		
P180 (μV)	0.139 (0.120)	−0.096 to 0.374	0.246		
**Amplitude**	**T2 vs. T3**				
	**Mean difference (μV) (SE)**	**95% CI for difference**	***p***		
P30 (μV)	−0.119 (0.112)	−0.339 to 0.101	0.288		
N45 (μV)	0.139 (0.112)	−0.080 to 0.358	0.214		
P60 (μV)	−0.117 (0.134)	−0.381 to 0.147	0.383		
N100 (μV)	0.056 (0.131)	−0.202 to 0.314	0.669		
P180 (μV)	−0.037 (0.121)	−0.274 to 0.200	0.758		
**Amplitude**	**BL vs. T3**				
	**Mean difference (μV) (S.E.)**	**95% CI for difference**	***p***		
P30 (μV)	−0.018 (0.112)	−0.238 to 0.201	0.870		
N45 (μV)	−0.018 (0.112)	−0.237 to 0.201	0.875		
P60 (μV)	0.177 (0.134)	−0.087 to 0.441	0.189		
N100 (μV)	−0.520 (0.130)	−0.778 to −0.262	< 0.001		
P180 (μV)	0.102 (0.121)	−0.096 to 0.374	0.399		

Regional analyses of TEP amplitudes across nine brain regions are presented in Table 2. Significant omnibus effects of time point (BL, T2, and T3) were observed for P30 in the MP region (*p* = 0.007); for N45 in the RF (*p* = 0.008), MC (*p* = 0.011), and RC (*p* = 0.001) regions; for P60 in the MC region (*p* = 0.012); for N100 in the LF (*p* = 0.002), MF (*p* < 0.001), RF (*p* = 0.002), MC (*p* < 0.001), and RC (*p* < 0.001) regions; and for P180 in the LF (*p* = 0.049), RF (*p* = 0.019), MC (*p* < 0.001), and MP (*p* < 0.001) regions. The patterns of change revealed by post hoc pairwise comparisons are described below.

**TABLE 2:** Differences in five TMS‐evoked EEG potentials (TEPs) in all nine brain regions: Left frontal (LF), mid‐frontal (MF), right frontal (RF), left central (LC), mid‐central (MC), right central (RC), left posterior (LP), mid‐posterior (MP), and right posterior (RP). First, overall differences between time points (TP), and second, paired comparisons between TPs (BL, T2, and T3). Only statistically significant values are presented, and paired comparisons are only presented when overall values for each TEP are significant.

TEP	Brain region	Mean amplitudes (μV) (SE) in TPs			F	Overall *p*‐value	Compared time points	Mean difference (μV) (SE)	95% CI for difference	Between TPs *p*‐value
		BL	T2	T3						
P30	MP	1.151 (0.159)	0.665 (0.159)	1.004 (0.16)	5.428	0.007	T2 vs. BL	0.486 (0.151)	0.184–0.788	0.002
							T3 vs. T2	−0.339 (0.153)	−0.644 to −0.034	0.030
N45	RF	−2.203 (0.413)	−1.378 (0.413)	−2.244 (0.416)	5.235	0.008	T2 vs. BL	−0.825 (0.301)	−1.426 to −0.224	0.008
							T3 vs. T2	0.866 (0.304)	0.258–1.473	0.006
	MC	−2.079 (0.234)	−2.565 (0.234)	−2.407 (0.235)	4.793	0.011	T2 vs. BL	0.486 (0.16)	0.166–0.805	0.003
	RC	−1.47 (0.211)	−0.902 (0.211)	−1.291 (0.212)	7.539	0.001	T2 vs. BL	−0.568 (0.15)	−0.867 to −0.269	< 0.001
							T3 vs. T2	0.389 (0.151)	0.088–0.691	0.012
P60	MC	1.232 (0.193)	0.854 (0.193)	0.811 (80.194)	4.780	0.012	T2 vs. BL	0.378 (0.149)	0.080–0.677	0.014
							T3 vs. BL	0.421 (0.151)	0.120–0.723	0.007
N100	LF	−3.428 (0.426)	−2.358 (0.426)	−2.494 (0.429)	6.752	0.002	T2 vs. BL	−1.07 (0.316)	−1.703 to −0.438	0.001
	MF	−3.166 (0.278)	−2.108 (0.278)	−2.175 (0.28)	10.965	< 0.001	T2 vs. BL	−1.058 (0.252)	−1.563 to −0.554	< 0.001
							T3 vs. BL	−0.991 (0.255)	−1.501 to −0.482	< 0.001
	RF	−4.138 (0.57)	−2.83 (0.57)	−3.063 (0.573)	6.662	0.002	T2 vs. BL	−1.308 (0.382)	−2.070 to −0.545	0.001
							T3 vs. BL	−1.075 (0.386)	−1.846 to −0.304	0.007
	MC	−2.59 (0.262)	−1.572 (0.262)	−1.739 (0.264)	13.839	< 0.001	T2 vs. BL	−1.018 (0.207)	−1.432 to −0.604	< 0.001
							T3 vs. BL	−0.852 (0.209)	−1.270 to −0.433	< 0.001
	RC	−2.573 (0.238)	−1.764 (0.238)	−1.896 (0.259)	10.228	< 0.001	T2 vs. BL	−0.809 (0.192)	−1.192 to −0,426	< 0.001
							T3 vs. BL	−0.677 (0.194)	−1.064 to −0.290	0.001
P180	LF	0.637 (0.168)	0.949 (0.168)	1.037 (0.17)	3.153	0.049	T3 vs. BL	−0.4 (0.168)	−0.735 to −0.065	0.020
	RF	0.931 (0.248)	1.312 (0.248)	1.511 (0.25)	4.232	0.019	T3 vs. BL	−0.58 (0.203)	−0.985 to −0.174	0.006
	MC	3.43 (0.283)	2.19 (0.283)	2.113 (0.285)	20.793	< 0.001	T2 vs. BL	1.24 (0.228)	0.784–0.352	< 0.001
							T3 vs. BL	1.317 (0.231)	0.856–1.778	< 0.001
	MP	0.984 (0.194)	0.367 (0.194)	0.408 (0.195)	9.948	< 0.001	T2 vs. BL	0.617 (0.154)	0.309–0.925	< 0.001
							T3 vs. BL	0.576 (0.156)	0.265–0.888	< 0.001

Post hoc pairwise comparisons revealed three distinct temporal patterns. First, several TEP component‐region combinations showed transient changes at T2 that recovered by T3. P30 amplitude in the MP region decreased significantly at T2 relative to BL (*p* = 0.002) and recovered by T3 (T3 vs. T2, *p* = 0.030), with no significant difference between BL and T3. N45 amplitude in the RF (T2 vs. BL, *p* = 0.008; T3 vs. T2, *p* = 0.006) and RC (T2 vs. BL, *p* < 0.001; T3 vs. T2, *p* = 0.012) regions attenuated at T2 and returned to baseline levels by T3. In contrast, N45 in the MC region showed a transient enhancement (increased amplitude) at T2 relative to BL (*p* = 0.003). N100 in the LF region was significantly attenuated at T2 (*p* = 0.001) but was no longer significantly different from BL at T3.

Second, several component–region combinations showed sustained changes that persisted through T3. P60 amplitude in the MC region decreased significantly at both T2 (*p* = 0.014) and T3 (*p* = 0.007) relative to BL. N100 showed the most widespread sustained effect, with amplitude remaining significantly attenuated at both T2 and T3 relative to BL in the MF (T2 vs. BL, *p* < 0.001; T3 vs. BL, *p* < 0.001), RF (*p* = 0.001; *p* = 0.007), MC (*p* < 0.001; *p* < 0.001), and RC (*p* < 0.001; *p* = 0.001) regions. P180 amplitude decreased at both T2 and T3 relative to BL in the MC (T2 vs. BL, *p* < 0.001; T3 vs. BL, *p* < 0.001) and MP (*p* < 0.001; *p* < 0.001) regions.

Third, P180 amplitude in the LF (T3 vs. BL, *p* = 0.020) and RF (T3 vs. BL, *p* = 0.006) regions increased significantly only at T3 relative to BL, without a significant change at T2, indicating delayed frontal enhancement. Overall, alcohol‐induced changes in regional TEP amplitudes were most prominent in frontal and central brain regions.

### 3.4 TEP Amplitudes and Clinical Correlations

There were no associations between clinical covariates such as AUDIT‐C score, BAC levels (at T2, T3, and Peak BAC of all measurements), lifetime psychiatric diagnosis, smoking, substance or illegal drug use, and TEPs at each time point, investigated with separate linear mixed models, standardized with brain regions.

### 3.5 Temporal and Spatial Dynamics of TEPs

**FIGURE 4:**
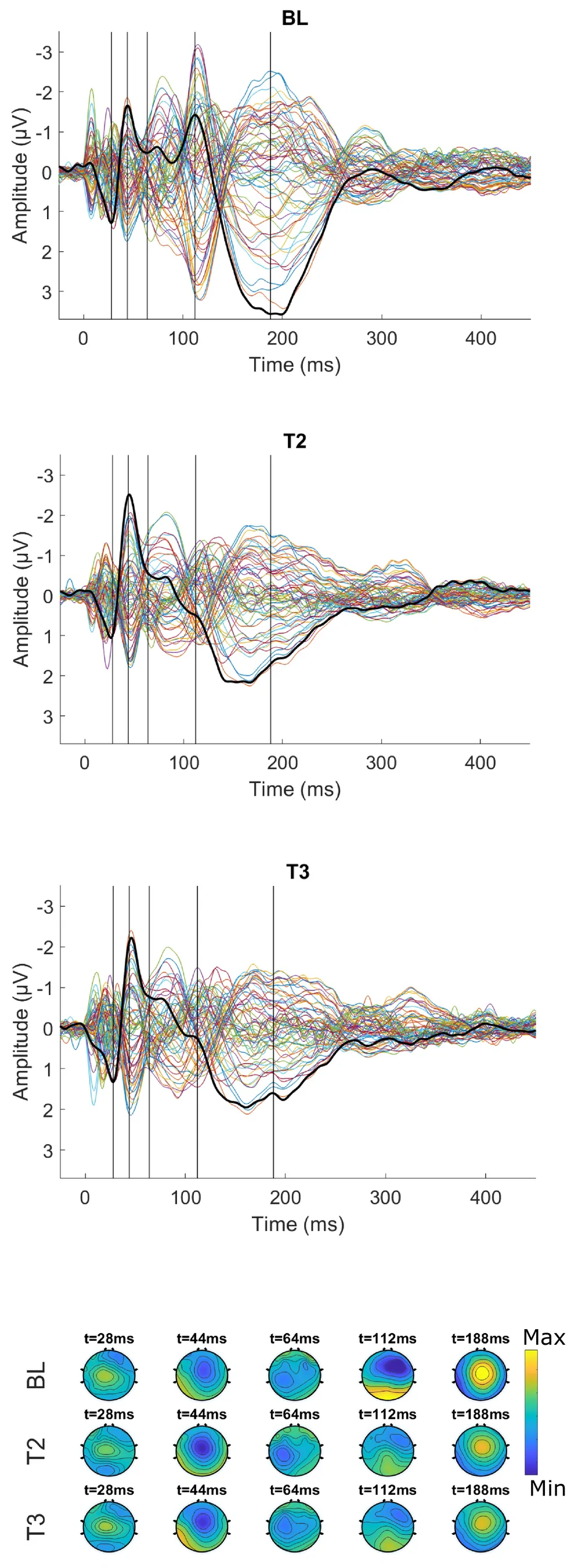
Butterfly and topographical plots of the transcranial magnetic stimulation‐evoked potential (TEP) peaks (μV) over time (ms) at BL, T2, and T3. Butterfly plots show the average in Cz (black line) and the average in each channel (colorful lines). Black horizontal lines in the butterfly plots reflect the time points at which the topographical plots were evaluated. These were the closest peaks to P30, N45, P60, N100, and P180. Data are presented from 33 participants who completed all time points (BL, T2, and T3).

TEP peaks were investigated through butterfly plots and scalp distribution maps (topographies) at BL, T2, and T3. Changes in the magnitudes of TEPs between BL and T2, and between BL and T3, were examined at 44 ms, 112 ms, and 188 ms. The topographies of the TEP peaks are presented in Figure 4.

## 4 Discussion

### 4.1 Summary of Key Findings

We investigated TMS‐evoked EEG responses after subthreshold motor cortex stimulation before alcohol intoxication (BL), at the estimated peak of the blood alcohol concentration (T2), and 120 min after alcohol consumption (T3). At the global level, only the N100 component showed a significant effect of time, with its amplitude attenuated (less negative) at both T2 and T3 compared to baseline, without recovery during the measurement period. No other components exhibited significant omnibus effects, although P60 showed a significant pairwise difference (less positive) at T2. Regional analyses revealed multiple component‐ and region‐specific changes, particularly in frontal and central areas. These changes followed three main temporal patterns: (1) transient effects, where amplitudes changed at T2 but returned to baseline by T3 (e.g., P30 in the MP region and N45 in the RF and RC regions); (2) sustained effects, where amplitudes remained altered at both T2 and T3 (most prominently N100 across several regions, as well as P60 and P180 in specific central areas); and (3) delayed effects, where changes emerged only at T3 (P180 in frontal regions).

Overall, the findings indicate that alcohol modulates TEP amplitudes in a temporally and regionally selective manner, with the most pronounced effects in frontal and central networks, and with the N100 component reflecting the most widespread and persistent neurophysiological alteration. However, as the analyses were exploratory in nature, the findings should be interpreted with caution.

### 4.2 Neurotransmitter‐Specific Modulation of TEPs

Alcohol modulates a variety of receptor systems and ion channels, including inhibition of NMDA and non‐NMDA glutamate receptors (Allgaier 2002; Zhao et al. 2016) and enhancement of GABA‐A receptor activity (Förstera et al. 2016). In addition, evidence summarized by Montemitro et al. (2025) indicates that both acute and chronic alcohol exposure may partly exert their neurophysiological effects through alterations in GABA‐B receptor–mediated inhibitory signaling (Montemitro et al. 2025). These neurochemical effects may be reflected in the modulation of specific TEP components, which in pharmacological TMS‐EEG studies have been associated with specific neurotransmitter systems P30 and P180 are associated with voltage‐gated sodium channel (VGSC) activity (Darmani et al. 2019), N45 with GABA‐A and NMDA receptor‐mediated glutamatergic transmission (Belardinelli et al. 2021; Premoli, Castellanos, et al. 2014), P60 with GABA‐A and AMPA receptor functioning (Belardinelli et al. 2021; Gordon et al. 2023), and N100 with GABA‐B receptor activity (Premoli, Castellanos, et al. 2014). However, although specific TEP components have been linked to particular neurotransmitter receptor systems, it should be noted that multiple neurophysiological processes are likely to contribute to their generation (Ziemann et al. 2026). In addition, it should be noted that late TEPs may be influenced by sensory and auditory artifacts rather than direct cortical activation (Freedberg et al. 2020).

Previous TMS‐EEG studies have interpreted alcohol‐induced attenuation of cortical responses as resulting from either a general reduction in network‐level excitability (Kähkönen et al. 2001, 2003; Kähkönen and Wilenius 2007) or a shift in neurotransmitter balance (Loheswaran et al. 2018). Specifically, reduced glutamate receptor activity combined with enhanced GABA‐A receptor‐mediated inhibition has been proposed to underlie suppression of the N100 component, which is sensitive to changes in inhibitory tone and excitatory drive (Loheswaran et al. 2018).

Our findings of sustained N100 attenuation during intoxication support these interpretations and address our study aims, suggesting that alcohol enhances slow inhibitory processes, particularly in the dorsolateral prefrontal cortex (DLPFC), consistent with PAS‐induced neuroplastic changes in the left DLPFC, reported by Loheswaran et al. (2017).

The paradoxical attenuation of N45 and N100, despite larger amplitudes typically indicating stronger GABAergic receptor activity, may be explained by reduced momentary glutamate availability for excitatory neurotransmission, which impairs the excitatory drive necessary for optimal inhibitory responses. Alcohol‐induced alterations in network‐level synchrony may further disrupt the generation or propagation of late TEPs. However, when linking individual TEP components to specific receptor systems, it is important to recognize that research on TEP components is still based primarily on experimental analyses.

### 4.3 Topographical and Temporal Divergence

We observed decreased GMFP post‐TMS during the estimated peak of the blood alcohol concentration at BL which supports a global reduction in cortical responsiveness at peak intoxication. There were also changes in the magnitudes of EEG signals at BL, emphasized at time windows for P30, N45, N100, and P180. In addition, we observed region‐specific increases in N45 and P180 components. P30 is a rather local response, and it has been used in assessing the learning of motor skills (Sasaki et al. 2024). However, the functional role of P30 remains incompletely understood. Our findings therefore contribute preliminary evidence on how early TEPs may behave under alcohol modulation. The enhancement of N45 in central regions may reflect increased GABA‐A receptor activity (Belardinelli et al. 2021; Förstera et al. 2016; Premoli, Castellanos, et al. 2014), suggesting that alcohol modulates inhibitory neurotransmission in a region‐specific manner, potentially reflecting compensatory cortical processes. Similarly, the frontal increase in P180 amplitude may indicate compensatory excitatory mechanisms or altered sensory integration under alcohol's influence.

Another notable observation was the differential recovery trajectory between early and late TEP components. Early components (e.g., P30, N45) tended to recover more rapidly than later ones (e.g., N100, P180). This may reflect their distinct origins: early TEPs are thought to arise from direct cortical activation by the TMS pulse, whereas later components may be more susceptible to sensory and acoustic artifacts (Freedberg et al. 2020; Gordon et al. 2023). Alternatively, the prolonged suppression of late components may be due to their association with GABA‐B receptor activity, supporting the notion that GABA‐B mechanisms are particularly relevant to alcohol‐induced neurophysiological alterations, potentially secondary to reduced glutamatergic drive and enhanced GABA‐A receptor activity.

Notably, acetate, a metabolite of alcohol, has been shown to enhance GABA synthesis (Goldman et al. 2025), potentially contributing to sustained inhibitory tone and delayed recovery of late TEPs. This temporal dissociation between early and late components is a relevant finding on recovery dynamics and suggests distinct underlying neurophysiological mechanisms.

### 4.4 Putative Receptor‐Specific Sensitivity and Implications

The differential modulation of P60 and N45 components may reflect alcohol's distinct effects on AMPA and NMDA receptor systems. P60 is associated with GABA‐A and AMPA receptor‐mediated excitatory transmission (Belardinelli et al. 2021; Gordon et al. 2023), while N45 is linked to NMDA receptor activity and GABA‐A‐mediated inhibition (Belardinelli et al. 2021; Premoli, Castellanos, et al. 2014). Alcohol inhibits NMDA receptors more potently than AMPA receptors (Allgaier 2002; Zhao et al. 2016), which may explain the more complex and regionally divergent changes observed in N45 compared to P60. This receptor‐specific sensitivity may also underlie the temporal differences in recovery: NMDA‐related processes (N45) may be more dynamically regulated and capable of partial rebound, whereas AMPA‐mediated transmission (P60) may remain persistently suppressed. These distinctions open avenues for future research investigating the contribution of excitatory and inhibitory neurotransmission to the cortical effects of alcohol and how they might be targeted in clinical or pharmacological interventions. However, although TEP components have been linked to specific neurotransmitter systems, they probably reflect composite network‐level activity rather than isolated receptor mechanisms.

We observed that alcohol consumption led to a decrease in GMFP. Alcohol intoxication‐induced reductions in prefrontal excitability (Kähkönen et al. 2003) may underlie impairments in cognitive performance and decision‐making (Jacob and Wang 2020). This suggests that alcohol reduces excitability in brain regions critical for higher cognitive functions (Kähkönen et al. 2003), representing a clinically relevant neurophysiological mechanism underlying cognitive impairment.

The N100 is a well‐established marker in TMS‐EEG studies, reflecting the balance between global cortical excitability and inhibition. Previous research (e.g., Premoli, Castellanos, et al. 2014; Zhou et al. 2022) has demonstrated that an increase in N100 amplitude may indicate increased GABAergic inhibition. Our findings of a sustained decrease in late TEPs, especially in N100 amplitude, during alcohol intoxication suggest that alcohol may enhance slow inhibitory processes, particularly in the dorsolateral prefrontal cortex (DLPFC). These findings are consistent with reported neuroplastic changes in the left DLPFC (Loheswaran et al. 2017). Similar alterations in N45 and P60 have been linked to GABA‐A activity and excitatory–inhibitory balance in psychiatric disorders (Fogel et al. 2024; Kähkönen and Wilenius 2007). Further research is needed to clarify the effects of acute alcohol intoxication on brain metabolism and the temporal dynamics of early TEPs. Together, these results provide converging evidence that acute alcohol intoxication preferentially alters slow inhibitory processes in prefrontal regions.

### 4.5 Limitations of the Study

While our findings are promising, we could not fully isolate the effects of alcohol from other factors such as alertness or individual tolerance. The participants were selected based on their previous alcohol consumption and with self‐rated alcohol use questionnaires, which must be considered when interpreting the results. Additionally, previous alcohol consumption may influence both subjective and neurophysiological responses to acute alcohol administration. There were no subjective or neurophysiological responses included in this study, which must be considered as a limitation. The interpretation of intoxication level relied on estimated BAC, but no subjective measures such as stimulation, sedation, or perceived intoxication, which limits the interpretation of the findings. The interpretation of TMS‐EEG data is complicated by individual variability and the complexity of EEG signals (Freedberg et al. 2020; Zhou et al. 2022). Although noise masking and a foam layer under the coil were used, the impact of sensory artifacts on later TEPs was possible. Moreover, interpreting TEP components across different brain regions requires more detailed spatial analysis. These challenges highlight the need for standardized protocols and controlled experimental designs. The regional findings are based on exploratory, uncorrected analyses and should therefore be interpreted with caution. The regional TEP analyses involved 135 pairwise comparisons without correction for multiple comparisons. At α = 0.05, approximately seven false positives would be expected by chance. Although the most robust findings, particularly the sustained N100 attenuation across multiple frontal and central regions (all *p* ≤ 0.001), are unlikely to reflect Type I error, effects with *p*‐values closer to 0.05 should be interpreted as exploratory and require replication.

### 4.6 Future Directions

While the effects of alcohol have been extensively studied in animal models and behavioral paradigms, TMS‐EEG now offers a safe and non‐invasive method to directly investigate alcohol‐induced cortical changes in humans. Importantly, future studies could explore dose‐dependent effects by systematically varying the blood alcohol concentrations. In our study, the estimated blood alcohol concentration probably reflected a moderate level, comparable to typical social drinking, but further research is needed to clarify how different doses influence cortical dynamics. Such investigations could provide valuable insights into the neural correlates of subtle cognitive impairments and help delineate the boundary between recreational use and neurophysiological disruption. For example, examining how TEP components respond to low versus moderate levels of intoxication could reveal thresholds at which specific neurophysiological changes occur. In addition, further research should investigate N100 changes over longer time frames and across varying levels of alcohol exposure. Combining TMS‐EEG data with behavioral and neuropsychological assessments would provide a more comprehensive understanding of alcohol's effects on cognition. Future studies should explicitly test the mechanisms suggested by our findings, particularly the role of excitation–inhibition imbalance and regional specificity. This would help translate neurophysiological findings into clinically meaningful markers of alcohol effects.

## 5 Conclusion

The neurophysiological functioning of the brain of healthy adults during alcohol intoxication was investigated with TMS‐EEG, and the exploratory study demonstrates time‐dependent and region‐specific modulation of cortical excitability during acute alcohol intoxication. Importantly, our findings highlight that alcohol's effects are dynamic, regionally heterogeneous, and not limited to simple global suppression. To our knowledge, this is the first study presenting early topographic changes in TEPs over time during acute alcohol intoxication. TMS‐EEG offers a promising, although still evolving, tool for assessing brain function and monitoring therapeutic effects. There are needs for a better understanding of the neurobiological underpinnings of these responses and their clinical significance. Further research is needed to fully understand their implications and to standardize the use of the method in clinical practice. Overall, TMS‐EEG opens new possibilities for investigating the acute effects of alcohol in humans, offering a translational bridge between preclinical findings and clinical relevance.

## Funding

The Youth Alcohol Project was funded by Government Research Funding, the Finnish Foundation for Alcohol Studies, Alko Inc., the Jenny and Antti Wihuri Foundation, the Instrumentariumin Tiedesäätiö, and the Finnish Cultural Foundation. Researcher Tommi Tolmunen was supported by the Strategic Research Council within the Academy of Finland (SchoolWell, grant number 352509, work package 352511).

## Conflicts of Interest

The authors declare no conflicts of interest.

## Data Availability

The data that support the findings of this study are available from the corresponding author upon reasonable request.
